# Insulin-inducible THRSP maintains mitochondrial function and regulates sphingolipid metabolism in human adipocytes

**DOI:** 10.1186/s10020-022-00496-3

**Published:** 2022-06-17

**Authors:** Maria A. Ahonen, Marcus Höring, Van Dien Nguyen, Sami Qadri, Juuso H. Taskinen, Meghana Nagaraj, Martin Wabitsch, Pamela Fischer-Posovszky, You Zhou, Gerhard Liebisch, P. A. Nidhina Haridas, Hannele Yki-Järvinen, Vesa M. Olkkonen

**Affiliations:** 1 Minerva Foundation Institute for Medical Research, Biomedicum 2U, Tukholmankatu 8, 00290 Helsinki, Finland; 2 Doctoral Programme in Clinical Research, University of Helsinki, Helsinki, Finland; 3 Institute of Clinical Chemistry and Laboratory Medicine, University Hospital Regensburg, Regensburg, Germany; 4 Division of Pediatric Endocrinology and Diabetes, Department of Pediatrics and Adolescent Medicine, University Medical Center Ulm, Ulm, Germany; 5 Systems Immunity University Research Institute, and Division of Infection and Immunity, Cardiff University, Cardiff, UK; 6 Department of Medicine, University of Helsinki and Helsinki University Hospital, Helsinki, Finland; 7 Department of Anatomy, Faculty of Medicine, University of Helsinki, Helsinki, Finland

**Keywords:** Hexosylceramide, Insulin sensitivity, Oxidation, Thyroid hormone

## Abstract

**Background:**

Thyroid hormone responsive protein (THRSP) is a lipogenic nuclear protein that is highly expressed in murine adipose tissue, but its role in humans remains unknown.

**Methods:**

We characterized the insulin regulation of THRSP in vivo in human adipose tissue biopsies and in vitro in Simpson-Golabi-Behmel syndrome (SGBS) adipocytes*.* To this end, we measured whole-body insulin sensitivity using the euglycemic insulin clamp technique in 36 subjects [age 40 ± 9 years, body mass index (BMI) 27.3 ± 5.0 kg/m^2^]. Adipose tissue biopsies were obtained at baseline and after 180 and 360 min of euglycemic hyperinsulinemia for measurement of *THRSP* mRNA concentrations. To identify functions affected by THRSP, we performed a transcriptomic analysis of THRSP-silenced SGBS adipocytes. Mitochondrial function was assessed by measuring mitochondrial respiration as well as oxidation and uptake of radiolabeled oleate and glucose. Lipid composition in THRSP silencing was studied by lipidomic analysis.

**Results:**

We found insulin to increase *THRSP* mRNA expression 5- and 8-fold after 180 and 360 min of in vivo euglycemic hyperinsulinemia. This induction was impaired in insulin-resistant subjects, and *THRSP* expression was closely correlated with whole-body insulin sensitivity. In vitro, insulin increased both THRSP mRNA and protein concentrations in SGBS adipocytes in a phosphoinositide 3-kinase (PI3K)-dependent manner. A transcriptomic analysis of THRSP-silenced adipocytes showed alterations in mitochondrial functions and pathways of lipid metabolism, which were corroborated by significantly impaired mitochondrial respiration and fatty acid oxidation. A lipidomic analysis revealed decreased hexosylceramide concentrations, supported by the transcript concentrations of enzymes regulating sphingolipid metabolism.

**Conclusions:**

THRSP is regulated by insulin both in vivo in human adipose tissue and in vitro in adipocytes, and its expression is downregulated by insulin resistance. As THRSP silencing decreases mitochondrial respiration and fatty acid oxidation, its downregulation in human adipose tissue could contribute to mitochondrial dysfunction. Furthermore, disturbed sphingolipid metabolism could add to metabolic dysfunction in obese adipose tissue.

**Supplementary Information:**

The online version contains supplementary material available at 10.1186/s10020-022-00496-3.

## Introduction

The thyroid hormone sensitive protein (THRSP; Spot14; S14) is a nuclear protein, which is abundantly expressed in lipogenic tissues such as in liver, mammary gland, and adipose tissue (AT) and lipogenic breast cancers (Freake and Moon 2003; Freake and Oppenheimer 1987; Jump 1989; Jump et al. 1984). In the rat liver, expression of THRSP is significantly induced by thyroid hormone stimulation (Jump 1989). Although THRSP was first characterized in 1981, data regarding its physiological functions remain inconclusive (Seelig et al. 1981). Zhu et al. showed that THRSP deletion in mice enhanced hepatic de novo lipogenesis, while Wu et al. reported a decrease in hepatic lipogenesis (Wu et al. 2013; Zhu et al. 2001). In whole-body knock-out mice, Anderson et al. reported a weight reduction, improved glucose tolerance, and enhanced insulin sensitivity, while several contradictory studies in animal models suggested a positive correlation between THRSP expression and insulin sensitivity, glucose tolerance, and lipid synthesis (Anderson et al. 2009; Cao et al. 2007; Wang et al. 2007, 2004).

Data are sparse regarding the role of THRSP in human metabolism. Serum and AT levels of the protein are decreased in subjects with the metabolic syndrome, while its expression is upregulated during adipogenic differentiation (Chen et al. 2019; Ortega et al. 2010). These findings suggest a functional role for THRSP in healthy AT expansion. Importantly, our group has previously found THRSP to be one of the top-regulated genes in micro array analysis of human AT upon infusion with insulin (Soronen et al. 2012). Whether THRSP acts as a mediator of insulin-regulated metabolic pathways in AT is unknown. In addition to lipid storage capacity, the composition of stored lipids affects adipocyte metabolism and signaling to other cell types (Ahonen et al. 2021; Leiria and Tseng 2020). There are, however, no studies showing whether THRSP modulates adipocyte lipid composition.

Mitochondrial dysfunction has a detrimental impact on adipocyte metabolism and is thought to contribute to the pathogenesis of obesity, insulin resistance, and type 2 diabetes (Lowell and Shulman 2005; Rocha et al. 2020; Van Der Kolk et al. 2021). Thyroid hormones impact the mitochondrial function by alterations of ATP synthesis, oxidative phosphorylation, fatty acid transport and mitochondria biogenesis (Harper and Seifert 2008; Sinha et al. 2018; Weitzel et al. 2003). However, the role of THRSP in mediating the mitochondrial effects of thyroid hormones is unclear.

As we hypothesized THRSP to be a likely regulator of adipocyte metabolism, we systematically assessed its functions in human AT in vivo and in a cultured human adipocyte model in vitro. Specifically, we wished to validate the induction of THRSP by insulin in human adipocytes and to determine whether THRSP is a potential mediator of lipogenic actions of insulin in humans. To this end, we studied *THRSP* expression in AT biopsies of 36 individuals, obtained during euglycemic hyperinsulinemia. We further replicated the analysis in cultured adipocytes and determined whether silencing of THRSP conferred transcriptional changes in central metabolic pathways. As this was found to be the case, we studied the effects of THRSP silencing on cellular mitochondrial functions and on the adipocyte lipidome.

## Materials and methods

### Subjects and design of the clinical study

We recruited a total of 36 non-diabetic volunteers based on the following inclusion criteria: (a) age 18–60 years; (b) a body mass index (BMI) ≤ 40 kg/m^2^; (c) no evidence of acute or chronic disease other than obesity based on history, physical examination, electrocardiogram, and standard laboratory tests (complete blood counts, serum creatinine, thyrotropin, and electrolyte concentrations); (d) no use of drugs potentially affecting glucose tolerance; and (e) not pregnant or lactating. All volunteers were women. Each subject underwent a history and physical examination, including measurement of body weight and height. Fasting blood samples were drawn for measurement of plasma glucose, serum insulin, and serum C-peptide concentrations. The percentage of body fat was determined by using a bioelectrical impedance analysis (BioElectrical Impedance Analyzer System Model #BIA-101A; RJL Systems, Detroit, MI, USA). Subcutaneous fat volume was determined using magnetic resonance imaging, as previously described in detail (Ryysy et al. 2000). Whole-body insulin sensitivity of each subject was determined using the euglycemic hyperinsulinemic clamp technique (DeFronzo et al. 1979; Westerbacka et al. 2006; Yki-Jarvinen et al. 1984). Briefly, we used a primed-continuous infusion of regular human insulin (Insulin Actrapid; Novo Nordisk, Denmark), with the continuous part of infusion given at a rate of 1 mU/kg·min for 6 h. Normoglycemia was maintained by adjusting the rate of a 20% glucose infusion, based on plasma glucose measurements sampled every 5 min from arterialized venous blood. Aspiration needle biopsies of subcutaneous AT (SAT) were obtained before hyperinsulinemia and at 180 and 360 min after the start of the infusion. The biopsies were immediately snap-frozen in liquid nitrogen and subsequently stored at − 80 °C until further analysis. Insulin sensitivity (M-value) of the subjects was calculated from the glucose infusion rate required to maintain normoglycemia from 30 to 360 min, and the median M-value was used to divide the subjects into insulin-sensitive (IS) and insulin-resistant (IR) groups (DeFronzo et al. 1979). Each participant provided a written informed consent after being explained the nature and potential risks of the study, which received approval from the Ethics Committee of the Hospital District of Helsinki and Uusimaa (Helsinki, Finland).

### Cell culture and transfections

We studied the function of THRSP by silencing or overexpressing the gene in human adipocytes. To silence THRSP, Simpson-Golabi-Behmel syndrome (SGBS) adipocytes were cultured and differentiated for 14 days (Fischer-Posovszky et al. 2008; Wabitsch et al. 2001). Mature adipocytes were then transfected with either 200 nM of THRSP siRNA (siRTHRSP; Ambion; AM16704, ID:12758) or Silencer Select™ non-targeting control 2 (SS2; Thermo Fisher Scientific 4390846, Waltham, MA), by using the RNAiMax™ transfection reagent (Thermo Fisher Scientific; 13778-150). Transfection complexes underwent incubation on the cells for 72 h, followed by lysing or further use for downstream experiments. Insulin induction of SGBS was performed by first starving the cells in serum-free low-glucose medium, and then treating with 100 nM insulin or insulin and 50 µM LY294002 (LY) overnight.

To overexpress THRSP in SGBS cells, the cells were cultured as specified above. Preadipocytes were then infected with lentiviral particles expressing THRSP (THRSP oex; accession number BC031989) or control particles generated from an empty pENTR2B vector (oex ctrl), along with polybrene (8 µg/ml). Transduction was carried out for 24 h, followed by switching to a serum-free medium. After 24 h, the medium was again replaced with a culture medium containing serum and blasticidin (20 µg/ml; Invitrogen; R210-01) for 2 days. Next, the cells were cultured and differentiated as specified above. The constructs were generated by the Genome Biology Unit, which is supported by HiLIFE and the Faculty of Medicine, University of Helsinki, and Biocenter Finland.

To further validate our observations made in human adipocytes, we repeated the key experiments in 3T3-L1 mouse adipocytes. The cells were differentiated as described previously (Mysore et al. 2017). Insulin induction was performed as specified above.

### RNA sequencing

After the transfection of mature SGBS adipocytes with either siRTHRSP or SS2 (see above), the cells were lysed and their RNA extracted using the RNeasy mini kit (Qiagen; 74104), followed by DNase I treatment, according to the manufacturer’s protocol. The library was prepared using the TruSeq Stranded mRNA kit (Illumina) according to manufacturer’s protocol. Sequencing was performed on the Illumina NovaSeq 6000 platform using 2 × 100 bp paired-end reads for analysis. Demultiplexing of the sequencing reads was performed with Illumina bcl2fastq version 2.20. Adapters were trimmed with Skewer version 0.2.2 (Jiang et al. 2014). The quality of FASTQ files was analyzed with FastQC version 0.11.5-cegat (Andrews 2010).

RNA-sequencing analysis was performed using the Chipster suite (Kallio et al. 2011) according to the following workflow: (1) FASTQ reads were trimmed using Trimmomatic (Bolger et al. 2014); (2) Trimmed pair-ended reads were aligned to the Homo_sapiens GRCh38.95 genome using STAR (Dobin et al. 2013); (3) Aligned reads were counted using HTSeq (Anders et al. 2015); (4) Differential expression analysis was performed using DESeq2 (Love et al. 2014); (5) Ensembl identifiers were annotated using BioMaRt (Durinck et al. 2009).

For the Reactome pathway analysis, the list of differentially expressed genes (DEGs) was first filtered by Entrez names. Of duplicate Entrez IDs, the most significantly differentially expressed ones were used for downstream analysis. Subsequently, the Reactome pathway database was employed for the interrogation using the ReactomePA package (Yu and He 2016). In order to compare pathways activated by either THRSP silencing or insulin stimulation, we used a previously published microarray dataset (GSE26637, downloaded from https://www.ncbi.nlm.nih.gov/geo/) of human SAT collected during an euglycemic hyperinsulinemic clamp (Soronen et al. 2012). We used the data of 5 arrays, each generated from SAT of lean insulin-sensitive females collected at fasting and at 180 min of sustained hyperinsulinemia. Raw data (CEL files) were normalized by using the Robust Multichip Average (RNA) normalization function of the affy package (Gautier et al. 2004). Next, to determine the transcriptomic alterations in SAT induced by hyperinsulinemia with reference to fasting, DEG analyses were performed using the limma algorithm (Phipson et al. 2016). The DEG results then underwent probe set annotations using their corresponding chips and filtering for duplicated genes, of which the most significant probe was retained. Pathway analyses were subsequently performed using the ReactomePA package (Yu and He 2016). Significantly altered pathways were further compared between insulin resistance and THRSP silencing, and only the commonly altered pathways were selected.

### Gene expression analysis

Quantitative real-time PCR (qPCR) was used to measure gene expression in the SGBS and 3T3-L1 adipocytes and human SAT. Total RNA from SGBS cells or from tissue biopsies was isolated using the Lipid Tissue Mini Kit (Qiagen; Gaithersburg, MD) according to the manufacturer’s protocols. RNA from 3T3-L1 cells was isolated using PureLink™ RNA Mini Kit (InVitrogen, Carlsbad, CA; 12183018A). The SuperScript® VILO™ synthesis Kit (Invitrogen, Carlsbad, CA; 11754-050) was used for reverse transcription of cDNA. To quantify mRNA expression, qPCR was performed using the Lightcycler® SYBR-Green® master mix (Roche Diagnostics, Mannheim, Germany; 04887352001) and a LightCycler 480 II Real-Time PCR system (Roche Applied Science, Penzberg, Germany). For analysis, crossing point (Cp) values were calculated from amplification curves and normalized to Cp values of the housekeeping genes ribosomal protein lateral stalk subunit P0 (36B4) and actin. Sequences of the used qPCR primers are listed in Table 1 and mouse qPCR primers are listed in Additional file 1: Table S5.

**Table 1:** Sequences of primers used for qPCR analysis

Primer name	Sequence
36B4 F	5′-CATGCTCAACATCTCCCCCTT-3′
36B4 R	5′-GGGAAGGTGTAATCCGTCTCC-3′
Actin F	5′-GACAGGATGCAGAAGGAGATT-3′
Actin R	5′-TGATCCACATCTGCTGGAAGG-3′
THRSP F	5′-CAGGTGCTAACCAAGCGTTAC-3′
THRSP R	5′-CAGAAGGCTGGGGATCATCA-3′

### Western blotting

SGBS and 3T3-L1 protein expressions were quantified by western blotting. Cells were lysed in RIPA buffer (15 mM Tris–HCl, pH 7.4, 1% NP40 1%, 1.25% sodium deoxycholate, 150 mM NaCl, 1 mM EDTA, 1% SDS). Equal amounts of protein were loaded on 10% SDS polyacrylamide gels (Fast Cast TGX Stain-Free, BioRad, Hercules, CA), and blotting was done on PVDF membranes using the BioRad Transblot system. The membranes were blocked and probed overnight with anti-THRSP (Proteintech; 13054-I-AP) in 5% milk in TBS, 0.5% Tween-20. Proteins were detected with enhanced chemiluminescence (Pierce ECL Western; Thermo Scientific, Waltham, MA; 32106). Image Lab (BioRad) was used to quantify the corresponding protein band intensities, which were normalized to total protein intensity.

### Measurement of mitochondrial respiration

The mitochondrial oxygen consumption rate (OCR) in control and THRSP-silenced or overexpressing SGBS cells was measured using the Seahorse XF96 Extracellular Flux Analyzer (Agilent Technologies). SGBS preadipocytes were plated onto XF96 cell culture plates (Agilent Technologies), differentiated, and transfected as described above. The cells were incubated for 1 h in XF base medium with 10 mM d-(+)glucose (Sigma; 68769), 1 mM sodium pyruvate (Sigma; S8636), and 2 mM l-glutamine (Gibco; 25030-024), in a CO_2_ free incubator. OCR was measured using the XF Cell Mito Stress Test Kit (Agilent Technologies) according to the manufacturer’s protocol. Maximal respiration rates were obtained and normalized to cell count. Hoechst (3.3 µM; Thermo Scientific; 62249) was used to stain the cells, and the counting was done using the Cytation 5 Cell Imaging Multi-Mode Reader (Biotek, Agilent Technologies). OCR in THRSP-overexpressing and control cells was measured in preadipocytes due to a defective differentiation capacity of the lentivirally transduced cells on Seahorse plates. The overexpressing cells were seeded onto XF96 cell culture plates and grown until confluency, followed by the OCR measurement.

### Measurement of fatty acid oxidation

The THRSP-silenced or overexpressing SGBS adipocytes were starved in a substrate-limited medium (Glucose-free DMEM [Gibco, 11966025]; 1 mM l-glutamine [Gibco, 25030-081]; 0.5 mM glucose) for 24 h. The next day, the cells were pre-treated for 3 h with 1 mM l-carnitine or 50 µM Etomoxir (EMD Millipore Corp. USA; 236020). Thereafter, the cells were incubated with [^3^H] oleic acid (0.1 µCi/well; Perkin Elmer; NET289005MC) and albumin-bound oleic acid Sigma; 03008) in KH buffer (25.0 mM NaHCO_3,_ 1.2 mM MgSO_4_ × 7H_2_O, 1.2 mM KH_2_PO_4,_ 4.7 mM KCl, 118.1 mM NaCl, 2.5 mM CaCl_2_ × 2H_2_O, 10 mM HEPES, pH7.4) for 2 h. The incubation medium was collected, and the samples were passed through OH^−^ ion exchange columns (Dowex 1X8-200 Ion Exchange Resin, 217,425, Merck). The flow-through was collected to scintillation vials, and the amount of oleate oxidized was determined from the radioactive water by liquid scintillation counting.

### Glucose uptake

The THRSP-silenced or overexpressing SGBS adipocytes were washed carefully with PBS and starved in glucose- and serum-free DMEM for 24 h. The cells were treated with 100 nM insulin for 20 min and then incubated with 50 nM deoxy-d-glucose and [^3^H] deoxy-d-glucose (0.5 µCi/well; Perkin Elmer; NET328A250UC) for 5 min. Glucose uptake was terminated by three washes with ice-cold PBS. The cells were lysed with 0.1% SDS and radioactivity was measured by liquid scintillation counting.

### Lipidomic analysis

Total lipids were extracted from THRSP-silenced and control mature SGBS adipocyte lysates by using the Bligh and Dyer method in the presence of naturally absent lipid species as internal standards (Bligh and Dyer 1959). The following lipid species were added as internal standards: cholesterol ester (CE) 17:0, CE 22:0, ceramide (Cer) 18:1;O2/14:0, Cer 18:1;O2/17:0, diglyceride (DG) 14:0/14:0, DG 20:0/20:0, free cholesterol (FC) [D7], lyso-phosphatidylcholine (LPC) 13:0/0:0, LPC 19:0/0:0, lyso-phosphatidylethanolamine (LPE) 13:0/0:0, PC 14:0/14:0, phosphatidylcholine (PC) 22:0/22:0, phosphatidylethanolamine (PE) 14:0/14:0, PE 20:0/20:0 (di-phytanoyl), phosphatidylglycerol (PG) 14:0/14:0, PG 20:0/20:0 (di-phytanoyl), phosphatidylinositol (PI) 17:0/17:0, phosphatidylserine (PS) 14:0/14:0, PS 20:0/20:0 (di-phytanoyl), sphingomyelin (SM) 18:1;O2/12:0, triglyceride (TG) 17:0/17:0/17:0, and TG 19:0/19:0/19:0. The lipid extract was recovered by a pipetting robot (Tecan Genesis RSP 150) and vacuum dried. The residues were dissolved in 7.5 mM ammonium acetate in methanol/chloroform (3:1, v/v) for low mass resolution tandem mass spectrometry and chloroform/methanol/2-propanol (1:2:4 v/v/v) with 7.5 mM ammonium formate for high resolution mass spectrometry.

The analysis of lipids was performed by direct flow injection analysis (FIA) using a triple quadrupole mass spectrometer (FIA-MS/MS; QQQ triple quadrupole) and a hybrid quadrupole-Orbitrap mass spectrometer (FIA-FTMS; high mass resolution). FIA-MS/MS (QQQ) was performed in positive ion mode using the analytical setup and strategy described previously (Liebisch et al. 2004). A fragment ion of *m/z* 184 was used for LPC (Liebisch et al. 2002). The following neutral losses were applied: PE and LPE 141, PS 185, PG 189 and PI 277 (Matyash et al. 2008). PE-based plasmalogens (PE P) were analyzed according to the principles described by Zemski-Berry (Zemski Berry and Murphy, 2004). Sphingosine based Cer and HexCer were analyzed using a fragment ion of *m/z* 264 (Liebisch et al. 1999). Quantification was achieved by calibration lines generated by addition of naturally occurring lipid species to the respective sample matrix.

A detailed description of the FIA-FTMS strategy was published recently (Höring et al. 2021, 2020). TG, DG, and CE were recorded as [M + NH_4_]^+^ in positive ion mode in range *m/z* 500–1000 for 1 min with a maximum injection time of 200 ms, an automated gain control of 1 × 10^6^, three microscans and a target resolution of 140,000 (at *m/z* 200). PC, phosphatidylcholine ether and SM were analyzed as [M + HCOO]^−^ in negative ion mode in range *m/z* 520–960 at the same resolution setting. Multiplexed acquisition was applied for the [M + NH_4_]^+^ of FC and the corresponding internal standard (FC[D7]) (Höring et al. 2019). Quantification was performed by multiplication of the spiked internal standard amount with analyte-to-internal standard ratio. Lipid species were annotated according to the latest proposal for shorthand notation of lipid structures that are derived from mass spectrometry (Liebisch et al. 2020).

### Statistical methods

Normality of the data was tested using the Shapiro Wilk’s test. The independent 2-sample 2-tailed Student’s t-test or the Mann–Whitney *U* test were used to compare two groups, whereas the one-way ANOVA or the Kruskal–Wallis test were employed to compare three or more groups of normally and non-normally distributed data, respectively. The Spearman’s correlation coefficient was used to determine bivariate correlations. Multiple Mann–Whitney *U* tests with FDR set to 5% were used to compare means of the lipidomic data. Data are in mean ± SD. A P value ≤ 0.05 was considered statistically significant. Statistical analyses were performed with GraphPad Prism 9.3.1 (GraphPad Software, Inc., La Jolla, CA, USA) or R 4.0.3.

## Results

### THRSP is induced by insulin in human adipocytes in vivo and in vitro

**Fig. 1:**
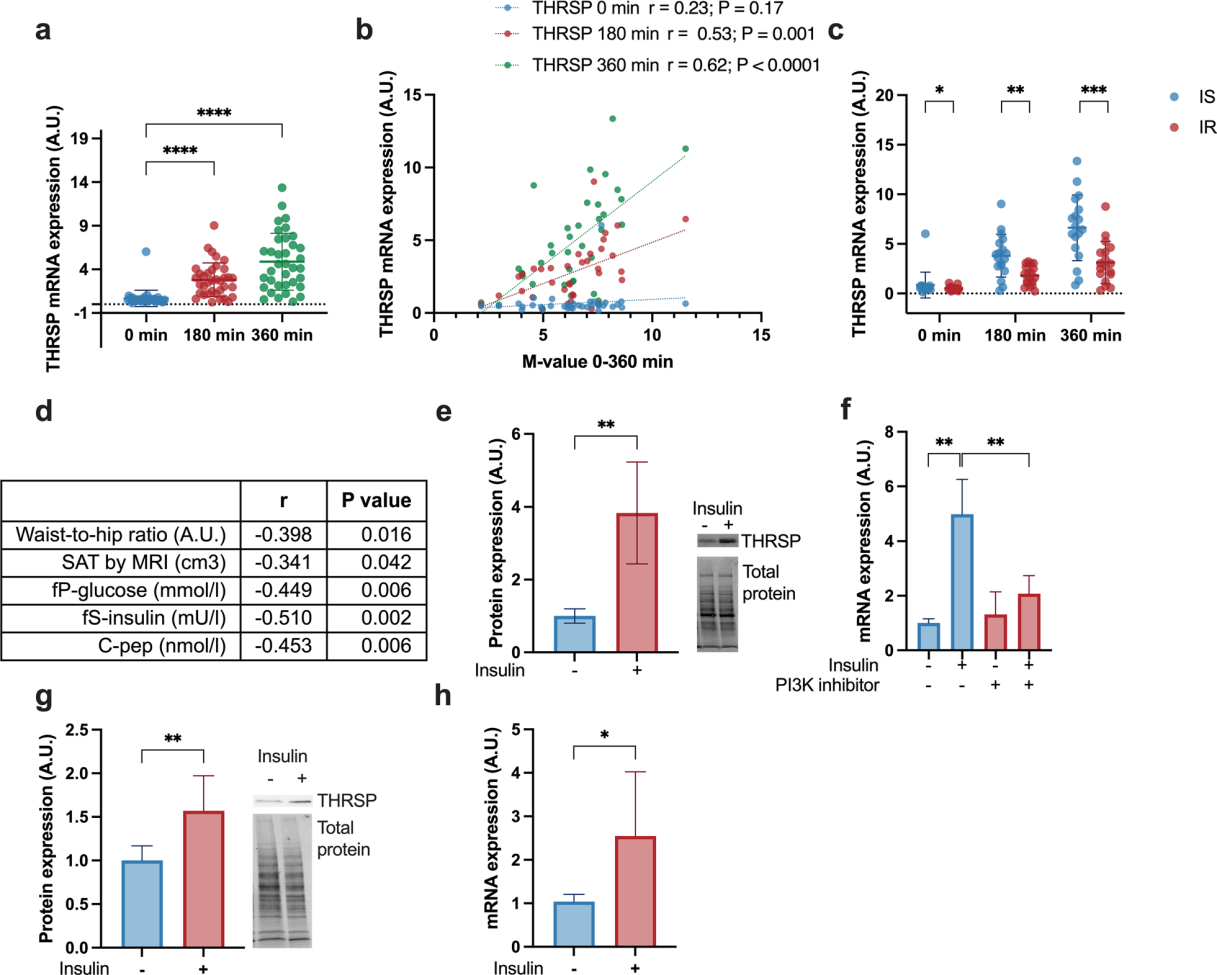
Insulin induces THRSP expression in human adipose tissue and human and mouse adipocytes. **a** Subcutaneous adipose tissue (SAT) biopsies were obtained in a euglycemic hyperinsulinemic clamp and THRSP mRNA expression was measured at basal, 180 min and 360 min (n = 36). **b** THRSP expression correlated with M-value, which is a measure of insulin sensitivity. The correlation was the strongest at 360 min. **c** Subjects were divided to insulin sensitive (IS; n = 18) and insulin resistant (IR; n = 18) group by median of M-value. THRSP induction by insulin was higher in IS group in comparison to IR group. **d** Correlation coefficients of waist-to-hip ratio, amount of SAT, fasting plasma glucose (fP-glucose), fasting serum insulin (fS-insulin) and C-peptide (C-pep) **e** Insulin induced THRSP protein expression in SGBS adipocytes (n = 6, three independent experiments). **f** LY294002, an inhibitor of PI3K, blocked insulin-mediated THRSP induction, measured at mRNA level (n = 6, two independent experiments). **g** Insulin induced THRSP protein expression in 3T3-L1 adipocytes (n = 10, three independent experiments). **h** Insulin induced THRSP mRNA expression in 3T3-L1 adipocytes (n = 5, two, independent experiments). The data is represented as mean with SD. Statistical significance is designated as *P ≤ 0.05 **P < 0.01, ***P < 0.001, ****P < 0.0001

Clinical characteristics of the subjects are shown in Table 2 (n = 36). Mean M-value for all subjects was 6.4 mg/kgBW/min, with SD of 1.8 mg/kgBW/min. Insulin induced *THRSP* expression in human SAT in a time-dependent manner (Fig. 1a). The induction by insulin was stronger in more insulin-sensitive subjects and *THRSP* expression correlated positively with the M-value, which is a measure of insulin sensitivity (r_360 min_ = 0.62; P < 0.0001; Fig. 1b, c). Moreover, clinical features associated with insulin resistance, such as the waist-to-hip ratio, SAT volume, serum insulin and C-peptide concentrations, and fasting plasma glucose concentrations, correlated inversely with *THRSP* expression (Fig. 1d).

**Table 2:** Clinical characteristics of the participants subjected to euglycemic hyperinsulinemic clamp

	All (n = 36)	IS (n = 18)	IR (n = 18)
Age (years)^a^	40 ± 9	38 ± 8	43 ± 11
Weight (kg)^a^	75 ± 15	69 ± 11	80 ± 16
BMI (kg/m^2^)^a^	27.3 ± 5.0	24.9 ± 3.7	29.8 ± 5.0
Postmenopausal (n)	6	1	5
Waist-to-hip ratio (A.U.)^a^	0.9 ± 0.1	0.8 ± 0.0	0.9 ± 0.1
fP-glucose^b^ (mmol/l)^a^	5.2 ± 0.6	5.0 ± 0.7	5.4 ± 0 ± 0.5
fS-insulin^c^ (mU/l)^a^	4.1 ± 1.6	3.5 ± 1.2	4.8 ± 1.7
M-value^d^ (mg/kgBW/min)^a^	6.4 ± 1.8	7.8 ± 1.1	5.0 ± 1.2
Body fat (%)^a^	32.9 ± 7.2	30.1 ± 7.2	35.7 ± 6.2
SAT by MRI (cm^3^)^a^	4111 ± 2189	2893 ± 1517	5328 ± 2103
C-peptide (nmol/l)^a^	0.58 ± + .18	0.51 ± 0.11	0.66 ± 0.20

^a^Data is represented as average ± SD

^b^fP, fasting plasma; ^c^fS, fasting serum; ^d^BW, body weight

To confirm that the insulin induction of *THRSP* occurs in adipocytes and also affects THRSP protein levels, mature human SGBS adipocytes were treated with insulin. Indeed, insulin significantly increased both THRSP mRNA and protein concentrations of the adipocytes (Fig. 1e, f). Addition of the PI3K inhibitor LY294002 (LY) abolished *THRSP* induction, suggesting that insulin regulates *THRSP* in a PI3K-dependent manner (Fig. 1f). THRSP induction by insulin was also confirmed in another cell model, 3T3-L1 adipocytes (Fig. 1g, h).

### THRSP silencing alters metabolic pathways

**Fig. 2:**
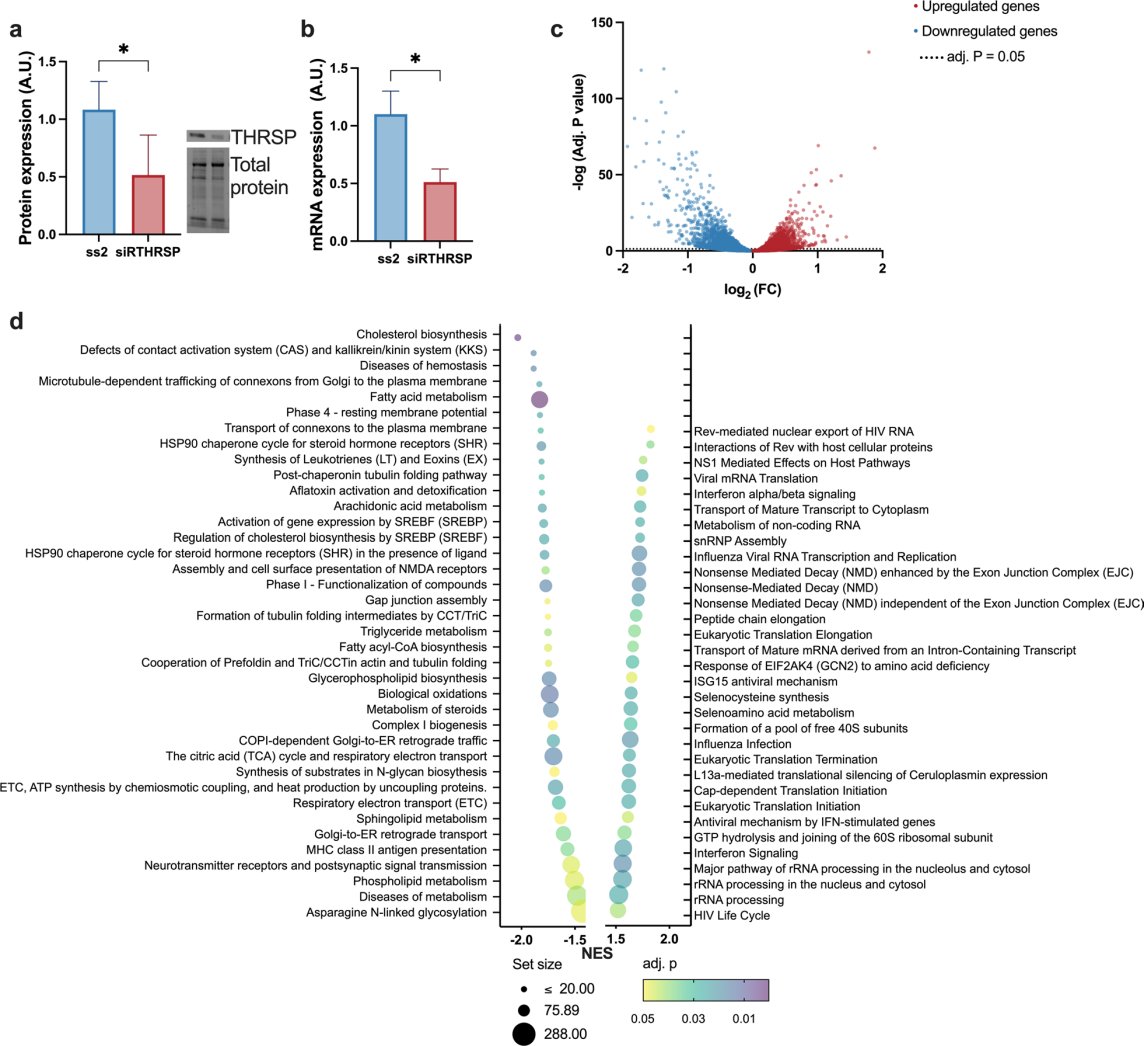
THRSP silencing alters adipocyte metabolism pathways in adipocytes. **a**, **b** To study the impact of low THRSP levels on adipocyte function and gene expression, adipocytes were transfected with non-targeting control siRNA (SS2) or THRSP siRNA (siRTHRSP; n = 5, two independent experiments). **c** RNA sequencing of the transfected cells revealed significant down regulation of 2124 genes and an increase of 2049 genes’ expression upon THRSP silencing (n = 4). **d** GSEA pathway analysis showed 38 significantly altered pathways with negative and 32 with positive NES score. The data is represented as mean with SD. Statistical significance is designated as *P ≤ 0.05 (**a**, **b**). The transcriptomic data with adjusted P value (P_adj_) ≤ 0.05 was considered as statistically significant

To identify putative functions of THRSP, we performed a transcriptomic analysis by next-generation RNA sequencing in THRSP-silenced SGBS adipocytes. Silencing efficiency was 52% at the protein level and 49% at the mRNA level (Fig. 2a, b). Of the over 27,000 total transcripts detected, those significantly differentially expressed numbered 4174 (P_adj_ ≤ 0.05; Fig. 2c). Expression data for the differentially expressed genes (DEGs) is shown in Additional file 2: Table S1. A gene set enrichment analysis (GSEA) revealed that multiple pathways involved in energy and lipid metabolism (cholesterol biosynthesis, fatty acid metabolism, citric acid cycle, steroid metabolism, and sphingolipid metabolism) had a negative normalized enrichment score (NES), indicating genes in those pathways to be mostly suppressed by THRSP silencing (Fig. 2d).

**Fig. 3:**
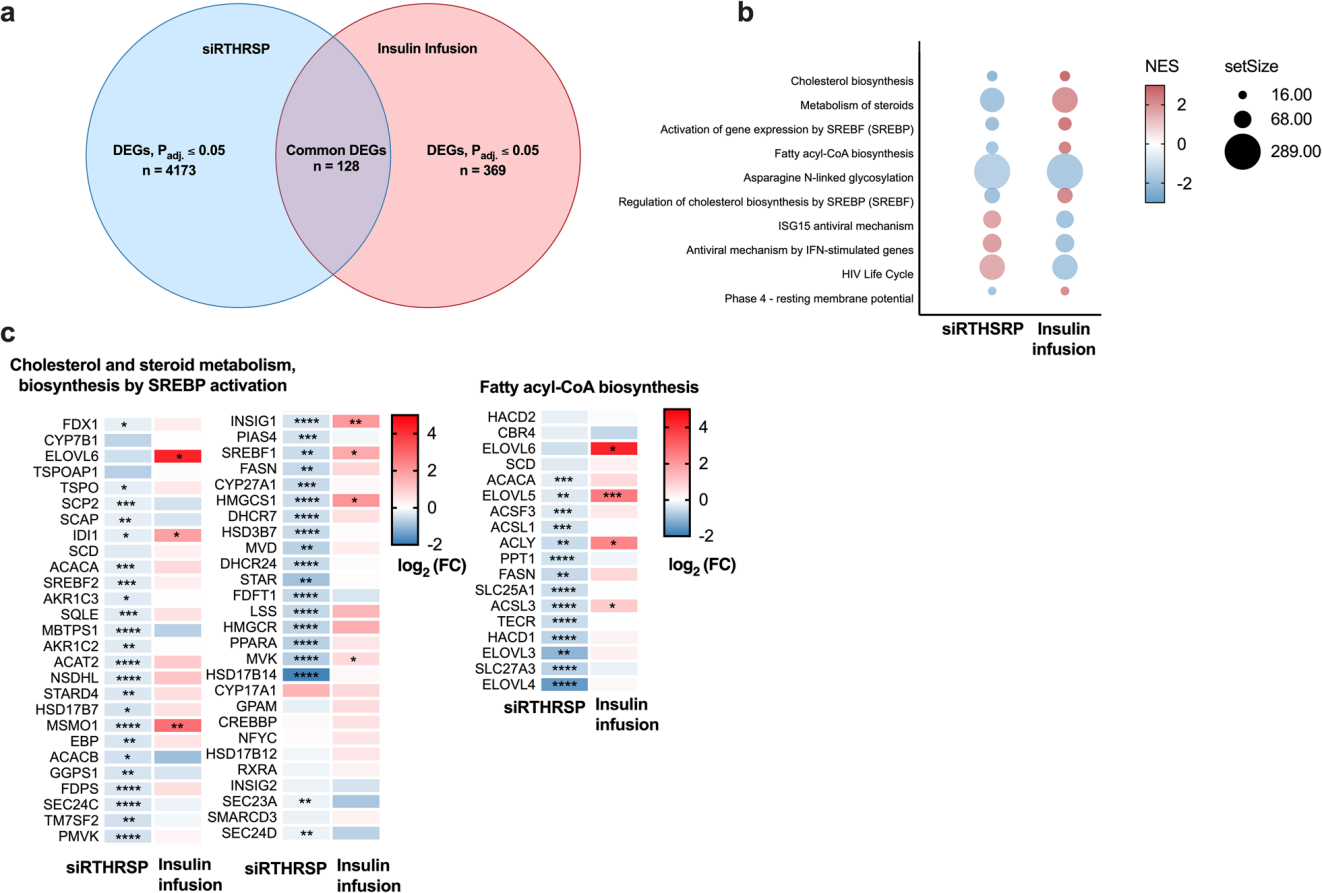
THRSP silencing and insulin targeted common pathways. **a** Venn diagram indicates the number of significantly altered genes in THRSP silencing in blue, hyperinsulinemia in red and overlapping significantly altered genes in the middle. **b** GSEA Reactome analysis was conducted from the transcriptome of human AT obtained during hyperinsulinemic clamp. Pathways affected by insulin infusion were compared to the pathways altered by THRSP silencing. There was 10 common pathways inversely altered. NES score is indicated by color and the set size by symbol size. **c** Significantly altered common metabolic pathways’ gene expression. Statistical significance is denoted as *P_adj_ ≤ 0.05, **P_adj_ < 0.01, ***P_adj_ < 0.001, ****P_adj_ < 0.0001

As we found *THRSP* to be an insulin-inducible gene, a further analysis was performed to understand which insulin-mediated functions in human SAT are altered in response to THRSP silencing. A publicly available microarray dataset (GSE26637) from our previous study was used to identify differentially expressed genes in AT during euglycemic hyperinsulinemia, and the altered gene profile was then compared with transcriptomic changes in the THRSP-silenced SGBS adipocytes. A total of 128 common DEGs were identified between the datasets (Fig. 3a; gene list in Additional file 1: Table S2). Several common and inversely affected pathways were identified by a Reactome GSEA (Fig. 3b). These included steroid metabolism, cholesterol biosynthesis, fatty acid biosynthesis, and SREBP-regulated pathways, suggesting that THRSP might be an important mediator of these functions of insulin in adipocytes. Gene expressions of the altered metabolic pathways are shown in Fig. 3c.

### THRSP silencing impairs mitochondrial function

**Fig. 4:**
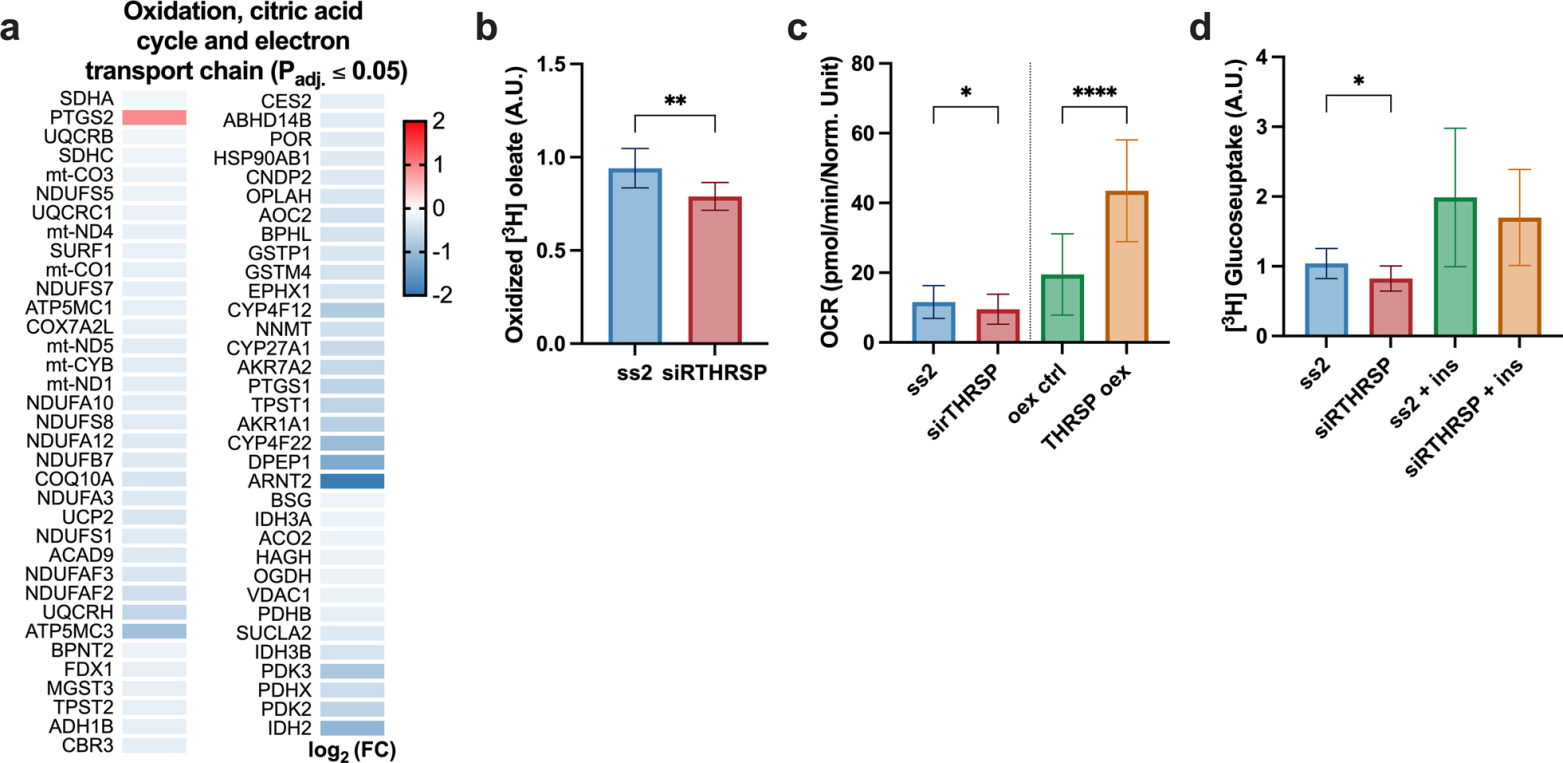
Mitochondrial function is dampened by THRSP silencing in adipocytes. **a** Oxidation, citric acid cycle and respiratory electron transport associated genes were affected by THRSP silencing. The heatmap represents log_2_ fold-change (log_2_ FC) of significantly altered genes (P_adj_ ≤ 0.05)*.* **b** Oxidation of [^3^H] oleate (n = 9, two independent experiments). **c** Oxygen consumption rate (OCR) measured in control (ss2), THRSP silencing (siR), overexpression control (oex ctrl) and overexpression (oex) (Two independent experiments with multiple replicates). d) Uptake of [^3^H] glucose in THRSP silenced adipocytes with or without insulin (n = 12, two independent experiments). Statistical significance is designated as *P ≤ 0.05 **P < 0.01, ****P < 0.0001

In THRSP-silenced adipocytes, genes involved in the citric acid cycle, oxidative phosphorylation, and oxidation were significantly downregulated (Fig. 4a, Additional file 1: Fig. S2c). Thus, we next assessed whether THRSP silencing affects mitochondrial functions. By employing radioactive oleate as a substrate, we found fatty acid oxidation to be significantly decreased in THRSP-silenced cells as compared to SS2 controls (Fig. 4b; P = 0.0037). To study mitochondrial respiration, the mitochondrial oxygen consumption rate (OCR) was measured using the Seahorse Mito Stress test. THRSP silencing in mature adipocytes led to significantly decreased maximal OCR (Fig. 4c; P = 0.035), whereas overexpression of THRSP in preadipocytes markedly increased OCR (Fig. 4c; < 0.0001). To determine whether the decreased mitochondrial activity is due to dampened mitochondrial biogenesis rather than mitochondrial function, we measured the ratio of mitochondrial DNA to genomic DNA. The ratio remained unchanged by THRSP silencing, implying that there was no inhibition of mitochondrial biogenesis (Additional file 1: Fig. S1).

To investigate if the dampened oxidative phosphorylation (as measured by OCR) is related to altered glucose uptake, we next measured the uptake of radioactive glucose in THRSP-silenced adipocytes. THRSP silencing decreased the uptake of glucose in comparison to SS2 controls (P = 0.03), but a similar change was not observable during treatment of the cells with insulin (Fig. 4d).

### THRSP silencing alters the lipid composition in adipocytes

**Fig. 5:**
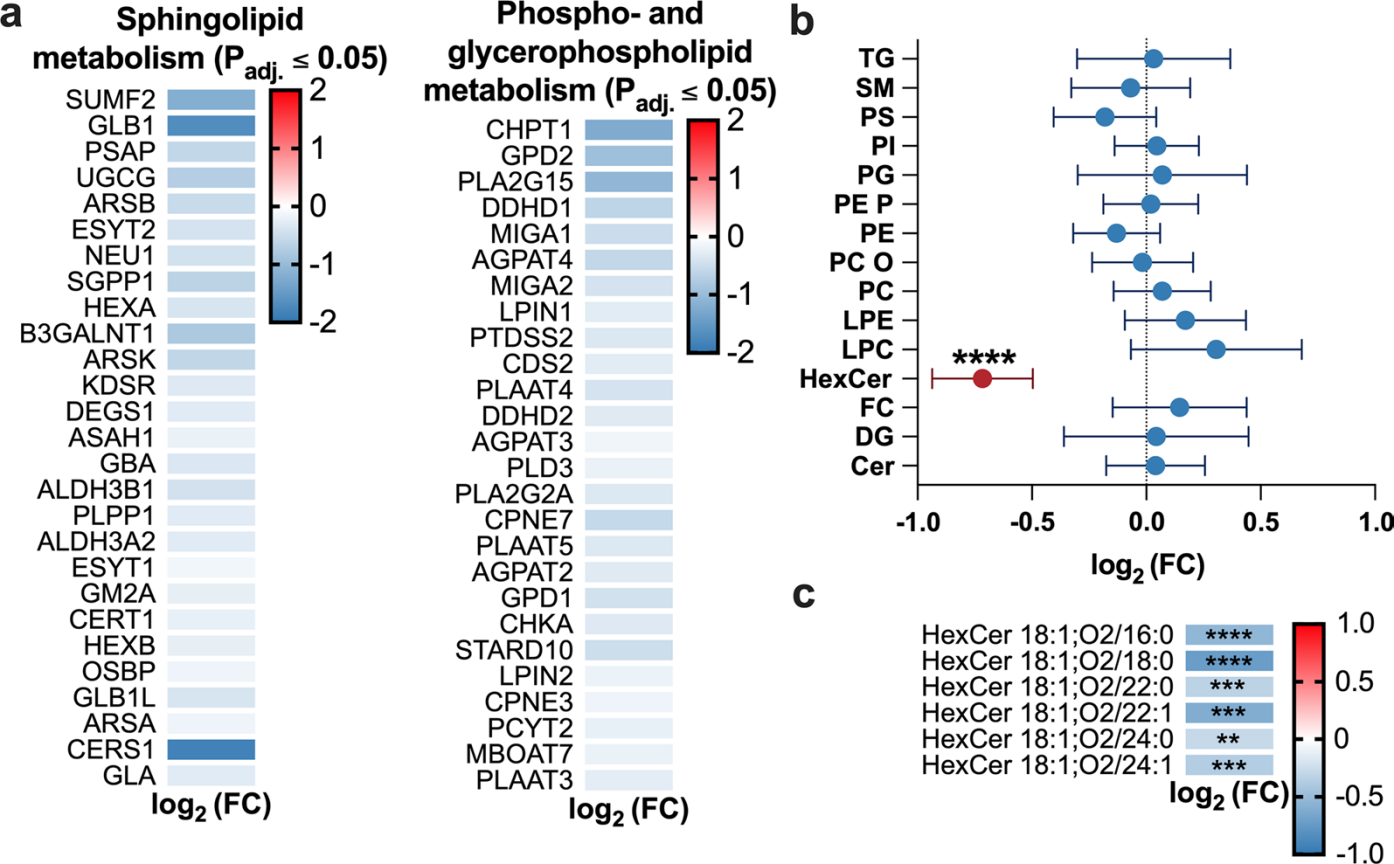
Adipocyte lipid composition is altered by THRSP silencing. **a** Significantly altered phospholipid, glycerophospholipid and sphingolipid metabolism genes and their log_2_ fold-changes (log_2_ FC; P_adj_ ≤ 0.05). **b** Log_2_ FC of lipid class concentrations between ss2 and sirTHRSP (n = 12, two independent experiments). **c** Log_2_ FC of hexosyl ceramide (HexCer) species. TG, triglycerides; DG, diglycerides; LPC, lyso-phosphatidylcholines; PE P, phosphatidylethanolamine plasmalogens; HexCer, hexosylceramides; Cer, ceramides; SM, sphingomyelins; PC O, phosphatidylcholine ethers; PC, phosphatidylcholines; PI, phosphatidylinositols; PG, phosphatidylglycerols; PS, phosphatidylserines; PE, phosphatidylethanolamines; LPE, lyso-phosphatidylethanolamines; FC, free cholesterol. Statistical significance is designated as **P_adj_ < 0.01, ***P_adj_ < 0.001, ****P_adj_ < 0.0001

**Fig. 6:**
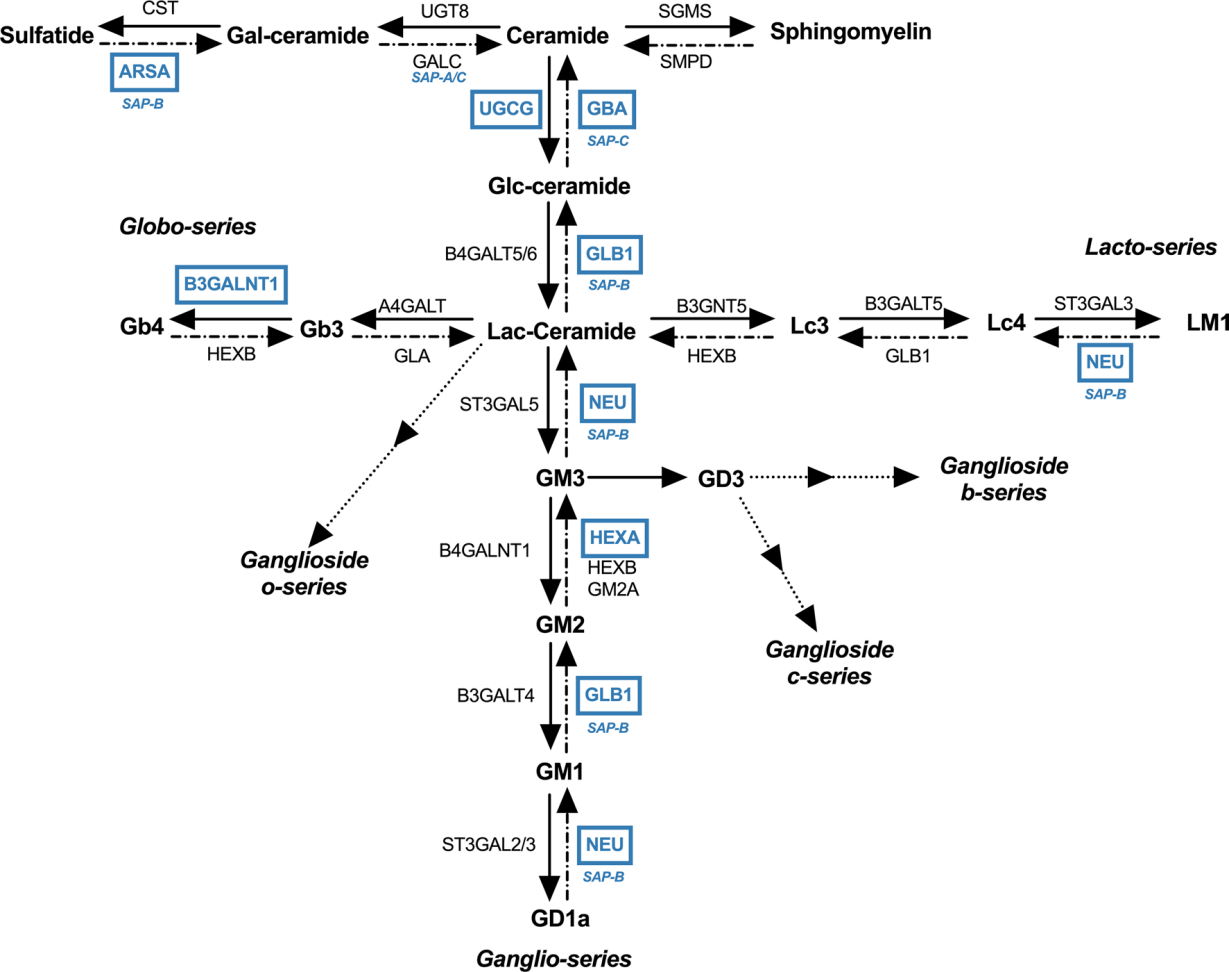
Schematic representation indicating sphingolipid metabolism genes downregulated in THRSP-silenced adipocytes. The figure represents sphingolipid metabolism, where the altered genes are designated by blue color. Saposin (SAP) below the gene name indicates activation by SAPs. GalCeramide, galactosylceramide; GlcCeramide, galactorylceramide; LacCeramide, lactosylceramide, CST, cystatin; ARSA, arylsulfatase A; SAP, saposin; UGT8, UDP glycosyltransferase 8; GALC, galactosylceramidase; SGMS, sphingomyelin synthase; SMDP, Sphingomyelin phosphodiesterase; UGCG, UDP-glucose ceramide glucosyltransferase; GBA, glucosylceramidase beta; B4GALT5/6, beta-1,4-galactosyltransferase 5/6; GLB1, galactosidase beta 1; B3GNT5, beta-1,3-N-acetylglucosaminyltransferase 5; B3GALT5, beta-1,3-galactosyltransferase 5; ST3GAL3, ST3 beta-galactoside alpha-2,3-sialyltransferase 3; NEU, neuraminidase; HEXB, hexosaminidase subunit beta; HEXA, hexosaminidase subunit alpha; GM2A, GM2 ganglioside activator; ST3GAL2/3, ST3 beta-galactoside alpha-2,3-sialyltransferase 2/3; B3GALT4, beta-1,3-galactosyltransferase 4; B4GALNT1, beta-1,4-N-acetyl-galactosaminyltransferase 1; ST3GALT5, ST3 beta-galactoside alpha-2,3-sialyltransferase 5; GLA, galactosidase alpha; B3GALNT1, beta-1,3-N-acetylgalactosaminyltransferase 1; A4GALT, alpha 1,4-galactosyltransferase

THRSP reportedly has a lipogenic function in various tissues, and the observed impairment of mitochondrial function during its silencing shows that THRSP regulates adipocyte energy metabolism (Freake and Oppenheimer 1987; Jump et al. 1984; Kinlaw et al. 1995). Moreover, the transcriptomic analysis suggested a potential role of THRSP in cholesterol, phospholipid, glycerophospholipid, and sphingolipid metabolism (Fig. 5a; cholesterol metabolism in Fig. 3c). We next performed a lipidomic analysis of THRSP-silenced adipocytes, which showed a significant reduction in the total concentrations of hexosylceramides (HexCer) compared to controls (Fig. 5b). Specifically, concentrations of HexCer species 18:1; O_2_/16:0, 18:1; O_2_/18:0, 18:1; O_2_/22:0, 18:1; O_2_/22:1, 18:1; O_2_/24:0, and 18:1; O_2_/24:1 were reduced (Fig. 5c, all lipid species shown in Additional file 1: Table S3). Corroborating these findings, the transcriptomic analysis revealed alterations in key genes regulating glycerophospho- and glycosphingolipid metabolism. In the glycosyl ceramide pathway, the key gene UDP-glucose ceramide glucosyltransferase (*UGCG*), which catalyzes the glycosylation of ceramides to glucosylceramides, was downregulated. Subsequently, downstream genes converting glucosylceramides to more complex glycosphingolipids were inhibited. Genes involved in the de novo synthesis of ceramides were also inhibited, including dihydroceramide desaturase (*DEGS1*) and ceramide synthases 1 and 4 (*CerS1*; -*4*; Fig. 6). Consistent with our observations in human adipocytes, genes involved in sphingolipid metabolism were downregulated in THRSP-silenced 3T3-L1 adipocytes as well (Additional file 1: Fig. S2c). Interestingly, when THRSP-silenced adipocytes with HexCer defect were treated with exogenous glucosylceramides, the mitochondrial respiration was restored (Additional file 1: Fig. S3).

## Discussion

In the present study, we investigated functions of the insulin-regulated gene product, THRSP, by employing human SAT biopsies and cultured human adipocytes. We studied the expression of THRSP before and during euglycemic hyperinsulinemia in vivo and found that insulin significantly induced THRSP expression, which was enhanced in subjects with high insulin sensitivity. A transcriptomic analysis of THRSP-silenced adipocytes revealed altered expression of genes not only related to lipid metabolism but also to mitochondrial function, oxidative phosphorylation, and oxidation pathways. Further analyses in cultured human SGBS adipocytes provided evidence for functions of THRSP in maintaining mitochondrial activity, fatty acid oxidation, and normal cellular sphingolipid concentrations, with a marked reduction of HexCer in THRSP-silenced adipocytes.

Our observations on the induction of THRSP by insulin are in agreement with the previously known lipogenic function of the protein (Freake and Oppenheimer 1987; Wu et al. 2013). A comparative transcriptomic analysis revealed that, in both THRSP-silenced adipocytes and insulin-stimulated AT, commonly affected genes belonged to the ‘Lipid metabolism’ and ‘SREBP-activated’ pathways—SREBP activity playing a crucial role in adipocyte lipogenesis (Gondret et al. 2001; Kim and Spiegelman, 1996). The above observations support a function of THRSP as a regulator of lipid homeostasis, while possibly mediating the effects of insulin on adipocyte lipid metabolism. Moreover, antiviral interferon-stimulated gene expression pathways were shared between adipocyte THRSP silencing and AT insulin induction in vivo. Interferon signaling in adipocytes is known to significantly affect adipocyte differentiation, lipogenesis, and immune responses (Lee et al. 2016; McGillicuddy et al. 2009; Wensveen et al. 2015). The downregulation of interferon-stimulated genes by insulin and THRSP might thus contribute to the enhanced insulin-mediated lipogenesis in adipocytes. However, a contradicting report claims that interferon signaling can improve metabolic dysfunction in AT (Wieser et al. 2018). Further studies are warranted to understand the insulin-mediated regulation of interferon signaling and how it regulates adipocyte lipid metabolism.

We observed quite a low number of pathways that were commonly regulated by insulin in AT in vivo and in adipocytes subjected to THRSP silencing. It is, however, worth noting that the method of differential expression analysis was different in the two datasets: next-generation RNA-seq was employed in the THRSP-silenced cells while microarrays were used in the in vivo study. This difference in technology might limit the number of pathways shared between the studies. On the other hand, the high number of genes and pathways affected by THRSP silencing may indicate that THRSP in adipocytes could execute additional insulin-independent functions that are crucial for AT metabolism. Therefore, a further effort was made to understand the functions of THRSP in adipocyte metabolism.

The RNA-seq and pathway analysis in THRSP-silenced cells revealed significant regulation of several mitochondrial functions, with alterations in key genes involved in oxidative phosphorylation and the TCA cycle (Fig. 4a). Mitochondrial function is generally dampened in obese AT, and this is often referred to as a hallmark of obesity (Schöttl et al. 2015; Vernochet et al. 2014). Of interest, a previous study has shown that THRSP expression is decreased in obesity (Ortega et al. 2010). Contrary to this finding, we did not observe a significant difference in THRSP expression between IS and IR subjects in the basal state, possibly due to the limited range of BMI among our study participants. Mitochondrial respiration was dampened, however, in adipocytes subjected to THRSP silencing. Despite the relatively low abundance of mitochondria in white adipocytes they are essential for the cells’ metabolic functions, and mitochondrial dysfunction does contribute to AT inflammation (Woo et al. 2019).

Thyroid hormone receptor signaling has been suggested to increase the trafficking of fatty acids into mitochondria (Sayre and Lechleiter, 2012). Since THRSP is also a T3-induced gene, it could be surmised to play a role in this process. Previous studies have shown that thyroid hormones induce enzymes favoring fatty acid oxidation, such as the uncoupling protein 2 (UCP2) (Sinha et al. 2018), which showed reduced expression in our THRSP-silenced adipocytes. Even though mitochondrial respiration and fatty acid oxidation was reduced upon THRSP silencing, the expression of CD36 was unaltered, and carnitine palmitoyltransferase 1A (CPT1a) expression was increased. However, the expression of fatty acid-binding proteins 3 and 7 (FABP3, -7) was decreased, suggesting an impaired influx of fatty acids into the cells, which putatively contributes to the reduced fatty acid oxidation. The observed reduction in OCR in the THRSP-silenced cells could also reflect a reduced glucose disposal rate (Woerle et al. 2003). While the reduction in OCR may in part be due to decreased glucose uptake, THRSP could also play a direct role in the regulation of oxidative phosphorylation, as suggested by our transcriptomic analysis. In alignment with our observations, THRSP was identified as a sirtuin 1-regulated gene in 3T3-L1 adipocytes, sirtuins being established regulators of mitochondrial function and biogenesis in human adipocytes (Majeed et al. 2021).

In addition to the direct effects on mitochondrial function, we observed that THRSP silencing significantly altered the adipocyte lipid composition. Several studies have shown that obesity and its metabolic consequences are closely related to disruptions in sphingolipid metabolism (Chaurasia et al. 2016; Green et al. 2021). Moreover, altered sphingolipid metabolism is connected to dysfunction of mitochondria (Knupp et al. 2017; Roszczyc-Owsiejczuk and Zabielski 2021). To this end, we observed a reduction in HexCer (glucosyl- and galactosylceramides) in the THRSP-silenced cells. Consistent with these changes, genes involved in the synthesis of glucosylceramides were downregulated (Fig. 6), including UDP-glucose ceramide glucosyltransferase (*UGCG*), the enzyme converting ceramide to glucosylceramide, as well as β-galactosidase (*GLB1*), neuraminidase 1 (*NEU1*), and hexosaminidase α (*HEXA*), which convert gangliosides back to glucosylceramide. In addition to reduced concentrations of HexCer, the downregulation of these enzymes could theoretically lead to an accumulation of ceramides and GM1, -2, and -3 gangliosides, all of which have been identified as contributors of insulin resistance in various cell types (Demir et al. 2020; Haynes et al. 2012; Kajihara et al. 2020; Lipina and Hundal, 2015; Sasaki et al. 2018; Wang et al. 2014; Yamashita et al. 2003). However, ceramide concentrations were not significantly increased, possibly due to the observed downregulation of ceramide synthases.

Interestingly, Chew et al. found HexCer plasma concentrations to negatively correlate with BMI and HOMA-IR, whereas ceramides showed a positive correlation (Chew et al. 2019). HexCer are known to play a role in resolving AT inflammation. They are presented by antigen-presenting cells as endogenous lipid antigens, resulting in iNKT cell activation and subsequent clearing of inflammation and the associated AT dysfunction (Lynch et al. 2012; Park et al. 2019; Rakhshandehroo et al. 2019; van Eijkeren et al. 2020, 2018). Although several publications have found sphingolipids as a whole to contribute to mitochondrial dysfunction, there is recent evidence that UGCG increases glycolysis and oxidative phosphorylation (Schömel et al. 2020). Supporting this finding, we observed that addition of exogenous glucosyl ceramide rescued the mitochondrial respiration in adipocytes subjected to THRSP silencing. Although addition of exogenous glucosylceramides does not rescue all of the alterations in lipid profile in THRSP-silenced adipocytes, it supports the notion that THRSP may impact mitochondrial functions both via the expression of genes involved in mitochondrial function and by regulating sphingolipid metabolism. Suppression of UGCG expression and the reduction in HexCer could thus be linked to impaired mitochondrial metabolism.

We also observed a reduction in the mRNA of several lysosomal hydrolases or their regulators, including arylsulfatase A, B, and K, choline phosphotransferase, and prosaposin. Prosaposin is a precursor of saposin, which is an activator of sulfatases in the sphingolipid metabolism. Deficiencies in these enzymes are linked to lysosomal storage disorders, but data is limited regarding their role in adipocyte metabolism or obesity (Allende et al. 2021; Monteith et al. 2016; Raj et al. 2020; Simonis et al. 2019; Tomanin et al. 2018; Trabszo et al. 2020). Lysosomal storage disorders are often associated with mitochondrial dysfunction, altered metabolism and fibrosis, showing parallels with obesity (Mizunoe et al. 2019; Pshezhetsky, 2015). Therefore, it might be of interest to study a possible role of THRSP in lysosomal storage diseases.

THRSP is regulated by insulin, thyroid hormone, carbohydrate intake, and fatty acids (LaFave et al. 2006). The present RNA-seq data suggest that, although insulin does induce THRSP, manipulation of either THRSP expression or insulin levels affected partly distinct pathways. In light of the present observations, we speculate that THRSP could execute distinct functions depending on the regulator that induces it. For instance, regulation of mitochondrial function could be a thyroid hormone-induced function of THRSP (Comas et al. 2019; Lanni et al. 2016). This is supported by the finding that mitochondrial pathways were not among the commonly regulated ones between AT insulin induction in vivo and the THRSP-silenced adipocytes. Moreover, non-coding RNA processing was identified as being potentially inhibited by THRSP, independent of insulin action. This aligns with previous research demonstrating that T3 inhibits various long non-coding RNAs in hepatocellular cancer—this inhibition could thus be mediated via THRSP (Huang et al. 2021).

Collectively, our findings shed light on the functions of THRSP in human AT in vivo and adipocytes in vitro, suggesting that THRSP plays a major role in the regulation of adipocyte metabolism via altering both mitochondrial function and cellular lipid composition. The present work adds to the existing knowledge, emphasizing the impact THRSP may have on whole-body health and development of metabolic disease. Fluctuations in THRSP expression might represent a physiologic response to nutritional and hormonal stimuli, which are known to be disturbed in obesity and metabolic disease. Thus, further studies are warranted to investigate whether restoring of THRSP responsiveness in insulin-resistant individuals could have therapeutic potential for ameliorating metabolic disease.

## Supplementary Information

**Additional file 1.** Supplementary methods, Table S2–S5, Fig. S1–S3.

**Additional file 2.** Table S1.

## Data Availability

Transcriptomic and lipidomic data of THRSP-silenced adipocytes are included in this article and its additional information files. Publicly available microarray data of SAT (GSE26637) can be downloaded from https://www.ncbi.nlm.nih.gov/geo/. Other data are available on reasonable request.

## Abbreviations

AT: Adipose tissue
BMI: Body mass index
Cer: Ceramides
DG: Diglycerides
DEG: Differentially expressed gene
FC: Free cholesterol
GSEA: Gene set enrichment analysis
HexCer: Hexosylceramides
LPC: Lyso-phosphatidylcholines
LPE: Lyso-phosphatidylethanolamines
NES: Normalized enrichment score
OCR: Oxygen consumption rate
PC: Phosphatidylcholines
PC O: Phosphatidylcholine ethers
PE: Phosphatidylethanolamines
PE P: Phosphatidylethanolamine plasmalogens
PG: Phosphatidylglycerols
PI: Phosphatidylinositols
PI3K: Phosphoinositide 3-kinase
PS: Phosphatidylserines
SAT: Subcutaneous adipose tissue
SGBS: Simpson-Golabi-Behmel syndrome (cells)
SM: Sphingomyelins
TG: Triglycerides
