# Supplementary material for: Insulin-inducible THRSP maintains mitochondrial function and regulates sphingolipid metabolism in human adipocytes
Source: Mol Med. 2022 Jun 17;28:68. doi: 10.1186/s10020-022-00496-3 (PMC9204892; doi:10.1186/s10020-022-00496-3)
Supplement: Supplementary file 1 — Additional file 1. Supplementary methods, Table S2–S5, Fig. S1–S3. [file 10020_2022_496_MOESM1_ESM.pdf]

1. Supplementary methods

a. Transfection of 3T3-L1 adipocytes

3T3-L1 adipocytes were transfected transfected with either 200 nM THRSP siRNA (siRTHRSP; Ambion; AM16708, ID:64998) or SS2 in serum-containing medium for 72 h.

b. Analysis of mtDNA

SGBS cells were differentiated and transfected as stated above. DNA was extracted from adipocyte lysates using a standard phenol-chloroform extraction method and precipitated by ethanol. Relative mitochondrial DNA (mtDNA) amount was quantified by performing qPCR as previously described (Heinonen et al., 2015). 16S RNA, CYTB and DLOOP expressions were employed as a measure of mtDNA and APP, B2M and HBB to measure genomic DNA (gDNA). The amount of mtDNA after THRSP silencing in comparison to the control was calculated as mtDNA/gDNA ratio. Primer sequences for mitochondrial DNA analysis are presented in Supplementary table 4.

c. Glucosylceramide feeding

SGBS cells were differentiated on Seahorse XF96 cell culture plates. Medium supplemented with 10µM glucosylceramide (C18 Glucosyl(ß) Ceramide (d18:1/18:0); 860547; Avanti Polar Lipids) and 1% lipoprotein deficient serum was sonicated and added onto cells overnight. Next day, the medium was replaced with the Seahorse assay medium and respiration measured as described in the methodology section.

Supplementary table 2. Common altered genes' $\log_2$ fold-change (FC) between THRSP silencing in SGBS adipocytes and adipose tissue upon insulin infusion. Genes with $P_{adj.} \leq 0.05$ in either or both conditions were included.

Gene symbol	$\log_2$FC in THRSP silencing	$\log_2$FC in insulin infusion	$P_{adj.}$ THRSP silencing	$P_{adj.}$ insulin infusion
PDK4	0.4831	-2.8647	0.0000	0.0000

SLC2A6	-0.2809	1.1889	0.0177	0.0000
MAT2A	0.1641	1.8731	0.0029	0.0000
OGA	-0.2007	-1.2939	0.0005	0.0001
ELOVL5	-0.3424	2.5994	0.0018	0.0002
PPTC7	-0.3595	1.1391	0.0015	0.0002
TEF	-0.6004	-1.6863	0.0000	0.0002
IRS2	-0.4300	-1.6011	0.0000	0.0003
GLCCI1	0.5598	-2.4849	0.0004	0.0003
HSPB8	-0.5119	1.2628	0.0000	0.0003
PANK3	-0.4274	1.7672	0.0000	0.0004
PPP1R3B	-0.8593	3.0818	0.0000	0.0004
EIF4EBP2	-0.7050	-1.8841	0.0000	0.0004
FGF2	0.2775	-1.7990	0.0012	0.0007
BHLHE40	-0.2282	2.7616	0.0324	0.0011
INSIG1	-0.4186	2.0074	0.0000	0.0016
MSMO1	-0.3597	2.8110	0.0000	0.0018
MID1IP1	-0.3472	1.6545	0.0003	0.0022
FAM160B1	0.2721	-1.3278	0.0002	0.0022
TMEM100	0.6085	-2.0278	0.0018	0.0033
CREBRF	-0.4205	-1.2783	0.0000	0.0036
ANGPTL4	0.9958	-2.1136	0.0000	0.0036
TWNK	0.3906	1.0680	0.0025	0.0041

ADAMTS12	0.6164	-0.9744	0.0000	0.0042
TBX15	0.1920	-1.0709	0.0354	0.0045
CDC14B	-1.0401	-1.4177	0.0000	0.0049
UBR2	0.2043	-0.9052	0.0111	0.0049
SLC25A44	-0.1833	1.0564	0.0459	0.0049
SLC31A2	-0.9200	-1.7596	0.0000	0.0052
CXXC5	-0.2739	1.5063	0.0001	0.0057
PCMTD1	0.3975	-0.9353	0.0000	0.0059
THRB	-0.3943	-1.5650	0.0137	0.0063
PHF3	0.2869	-1.2512	0.0061	0.0070
AGT	-0.3503	1.4179	0.0220	0.0083
URB2	0.3457	1.7621	0.0113	0.0083
CTPS1	0.5509	1.3070	0.0000	0.0086
SH3RF3	0.3994	1.5950	0.0008	0.0086
FILIP1L	0.4438	-1.3178	0.0000	0.0087
LIMA1	0.2518	-0.7755	0.0000	0.0090
DIRC3	-0.6329	-0.6696	0.0000	0.0101
PRUNE2	-0.2726	3.6756	0.0137	0.0104
IDI1	-0.2752	1.8703	0.0199	0.0105
SREBF1	-0.5242	1.6617	0.0054	0.0107
CDKN1A	-0.5459	1.5506	0.0000	0.0107
ERMP1	0.6424	-1.6606	0.0000	0.0110

SPATA7	-0.3850	-1.7343	0.0175	0.0110
ACSL3	-0.4920	1.0029	0.0000	0.0120
DDIT4	-0.2976	-2.2456	0.0002	0.0127
ACVR1	0.1894	-1.2892	0.0277	0.0127
JOSD1	-0.3553	-1.0862	0.0000	0.0130
HMGCS1	-0.5051	2.0572	0.0000	0.0132
JMY	0.3540	-0.5017	0.0001	0.0132
SMO	-0.3272	-0.6744	0.0035	0.0132
THRSP	-0.9052	1.5262	0.0002	0.0136
NFX1	0.1796	-0.9126	0.0225	0.0149
TP53INP1	-0.4536	0.8942	0.0000	0.0150
SIN3A	0.2121	-1.2696	0.0042	0.0152
IREB2	-0.6109	-0.4791	0.0000	0.0158
MOCS1	-0.5518	-0.9140	0.0000	0.0163
PSPC1	-0.2630	1.2684	0.0104	0.0164
FERMT2	0.3930	-0.7140	0.0000	0.0165
ZBTB37	0.3191	-0.8150	0.0140	0.0165
ADAM19	-1.3997	0.8207	0.0000	0.0166
KLHL24	-0.4659	-1.7677	0.0000	0.0166
ANKRD10	-0.2647	-0.8754	0.0064	0.0186
MYC	0.3864	1.3933	0.0000	0.0196
CHSY1	0.2634	1.1424	0.0028	0.0204

RASD1	-0.4191	-1.8169	0.0063	0.0208
ANKRD28	0.2066	-1.4434	0.0358	0.0209
UBC	-0.1927	-1.0082	0.0069	0.0237
STRIP1	0.3253	-0.6450	0.0000	0.0245
F3	0.6422	-1.0693	0.0062	0.0250
PARP16	0.3631	-0.6767	0.0459	0.0256
SPTLC2	0.3688	-0.7298	0.0000	0.0257
KLF10	0.2271	1.1965	0.0218	0.0263
NFATC3	-0.2859	-1.0750	0.0198	0.0272
LRP6	-0.3379	-0.8784	0.0000	0.0277
TSLP	0.4679	-1.2961	0.0332	0.0278
ACLY	-0.5310	2.4107	0.0064	0.0280
FBXO32	-0.5685	-1.2937	0.0000	0.0304
NCOR1	0.1842	-0.6185	0.0086	0.0309
CHAC1	0.3686	0.8933	0.0074	0.0310
TTBK2	0.3862	-0.6933	0.0001	0.0310
PPP1R9A	-0.5678	-1.1868	0.0242	0.0314
RSF1	0.2180	-1.0524	0.0064	0.0315
PPIF	-0.3416	1.9355	0.0005	0.0322
C8orf33	0.4588	0.7458	0.0000	0.0325
PPP2R1B	-0.3760	-1.0723	0.0000	0.0330
FOXJ2	0.3595	-0.8843	0.0000	0.0330

ABCA6	0.4296	-1.5516	0.0000	0.0331
AKAP12	-0.4358	-0.8541	0.0000	0.0331
FOSL2	-0.2877	-0.3941	0.0000	0.0334
TANGO6	-0.4727	-0.5386	0.0000	0.0336
DDX21	0.4622	0.7658	0.0000	0.0337
PXDN	0.2596	-0.6819	0.0000	0.0337
ANKIB1	0.2498	-0.8512	0.0008	0.0340
ARHGEF37	-0.6733	-0.9892	0.0018	0.0349
PLIN2	-0.1793	1.0790	0.0158	0.0351
SPIDR	0.2043	-0.8031	0.0111	0.0359
RAB42	-1.0223	0.6497	0.0003	0.0363
ARMCX2	-0.2731	-1.5645	0.0010	0.0363
UNG	-0.3748	-1.0760	0.0000	0.0363
CEP57	0.7376	-1.1301	0.0000	0.0364
SP1	0.2346	-0.7736	0.0004	0.0397
MAMDC4	0.8086	0.6706	0.0000	0.0397
SHPRH	0.3496	-1.4183	0.0006	0.0402
SH3GLB1	0.2394	-0.6406	0.0000	0.0402
NOP16	0.2655	1.1681	0.0102	0.0403
CFAP410	-0.4510	0.9824	0.0015	0.0403
RIOK3	0.1887	-0.7524	0.0265	0.0408
FRMD4A	0.3921	-1.3050	0.0000	0.0414

ARMCX1	-0.9835	-0.8663	0.0000	0.0417
GPD2	-0.9380	1.3464	0.0000	0.0423
SUN1	0.1407	-0.7285	0.0147	0.0424
MVK	-0.7310	0.7064	0.0000	0.0424
S100PBP	-0.5210	-0.6800	0.0000	0.0426
RAB11FIP1	-0.6714	-1.1936	0.0345	0.0432
FGF18	-1.9070	1.1284	0.0090	0.0439
MAST4	-0.5256	-1.0712	0.0000	0.0439
GYG2	-0.3267	-1.2575	0.0125	0.0442
MAPKBP1	0.2422	0.4709	0.0132	0.0442
RBM25	0.2037	-1.1092	0.0040	0.0442
TUG1	-0.2077	-0.8819	0.0205	0.0442
SMARCA1	-0.1591	-0.7225	0.0404	0.0460
RFC1	0.2333	-0.6825	0.0045	0.0473
MAP2K6	-0.5727	1.3078	0.0002	0.0477
IST1	-0.1312	-0.5055	0.0278	0.0485
PLEKHM3	0.2852	-0.7366	0.0086	0.0487

Supplementary table 3. Log_2 fold-changes (FC) of all individual lipid species. $P_{adj.} \leq 0.05$ were considered as statistically significant.

Species	$P_{adj.}$	Log_2FC
Cer 18:1;O2/16:0	0.9587	-0.0926
Cer 18:1;O2/18:0	0.8655	0.0227
Cer 18:1;O2/22:0	0.8524	0.0511
Cer 18:1;O2/22:1	0.8655	0.0269
Cer 18:1;O2/23:0	0.7954	0.0366
Cer 18:1;O2/24:0	0.5746	0.1511
Cer 18:1;O2/24:1	0.5790	0.1093
Cer 18:1;O2/26:1	0.8605	0.0965
DG 32:1	0.8655	0.0893
DG 34:1	0.8786	0.0902
DG 34:2	0.8655	0.1259
DG 36:1	0.7082	0.3823
DG 36:2	0.9587	0.0679
DG 38:2	0.6257	-1.1957
DG 38:3	>0.999999	0.1451
DG 38:4	0.5746	-1.4144
FC	0.6129	0.1731
HexCer 18:1;O2/16:0	0.0001	-1.1210

HexCer 18:1;O2/18:0	0.0007	-1.8745
HexCer 18:1;O2/22:0	0.0001	-0.5765
HexCer 18:1;O2/22:1	0.0007	-1.3608
HexCer 18:1;O2/24:0	0.0073	-0.4861
HexCer 18:1;O2/24:1	0.0010	-0.6709
LPC 16:0	0.4027	0.3604
LPC 16:1	0.5228	0.2154
LPC 18:0	0.2261	0.5259
LPC 18:1	0.4027	0.3323
LPC 18:2	0.7244	0.3261
LPE 16:0	0.4027	0.2962
LPE 16:1	0.7244	0.1698
LPE 18:0	0.1605	0.6532
LPE 18:1	0.5389	0.2298
LPE 20:1	0.5389	0.1609
LPE 20:3	0.8655	0.0586
LPE 20:4	0.4845	-0.6755
PC 30:0	0.5228	-0.3850
PC 30:1	0.9587	-0.1005
PC 32:0	0.0447	-0.5253
PC 32:1	0.4266	0.1511
PC 32:2	0.8786	0.0270

PC 34:1	0.4045	0.1487
PC 34:2	0.9154	0.0328
PC 34:3	0.9587	-0.0616
PC 36:1	0.5746	0.1221
PC 36:2	0.8655	0.0628
PC 36:3	0.8680	0.0913
PC 36:4	0.9841	0.0307
PC 38:2	0.9841	-0.0565
PC 38:3	0.9841	-0.0231
PC 38:4	0.9154	-0.0141
PC 38:5	0.7244	0.1177
PC 38:6	0.9841	0.0376
PC O-32:0	0.0506	-0.3572
PC O-32:1	0.9009	0.0351
PC O-34:1	0.9841	-0.0156
PC O-34:2	0.7082	0.1257
PC O-36:1	0.8765	0.0057
PC O-36:2	0.8655	0.0833
PC O-36:4	0.7143	0.0771
PC O-38:4	0.6779	-0.2415
PC O-38:5	0.4266	-0.4037
PE 32:1	0.5270	0.1543

PE 34:1	0.7535	0.0653
PE 34:2	0.9154	-0.0034
PE 36:1	0.9154	-0.1087
PE 36:2	0.8524	-0.1805
PE 36:3	0.9841	-0.0036
PE 36:4	0.8655	0.0514
PE 38:1	0.8655	-0.1903
PE 38:2	0.4027	-0.2768
PE 38:3	0.3651	-0.2492
PE 38:4	0.4045	-0.2269
PE 38:5	0.4045	-0.2953
PE 38:6	0.8459	-0.1096
PE 40:3	0.5746	-0.2625
PE 40:4	0.0506	-0.4004
PE 40:5	0.2515	-0.3599
PE 40:6	0.0500	-0.4896
PE P-18:0/22:3	0.9154	-0.1120
PE P-18:0/22:4	0.9154	-0.0018
PE P-18:0/22:5	0.4845	-0.3139
PE P-18:0/22:6	0.9154	-0.1405
PE P-18:1/16:0	0.8655	-0.1560
PE P-18:1/16:1	0.4045	0.1883

PE P-18:1/18:1	0.4845	0.1138
PE P-18:1/18:2	0.8524	0.0933
PE P-18:1/18:3	0.8786	-0.0674
PE P-18:1/20:3	0.8680	-0.1143
PE P-18:1/20:4	0.4266	-0.2760
PE P-18:1/22:3	0.6129	-0.2147
PE P-18:1/22:4	0.9154	-0.0609
PE P-18:1/22:5	0.9405	-0.1654
PE P-18:1/22:6	0.4045	-0.3171
PG 32:1	0.8655	0.1544
PG 34:1	0.8655	0.1246
PG 36:1	0.8655	0.0967
PG 36:2	0.9154	0.0586
PI 34:1	0.7535	0.1030
PI 34:2	0.9319	0.0320
PI 36:1	0.9154	-0.1611
PI 36:2	0.6129	0.1271
PI 36:3	0.1352	0.3011
PI 38:2	0.8765	0.0170
PI 38:3	>0.999999	-0.0398
PI 38:4	0.5790	0.1312
PI 38:5	0.4045	0.2674

PI 40:3	0.8765	0.0138
PI 40:4	0.9154	0.1395
PI 40:5	0.8655	0.0570
PI 40:6	0.9533	-0.1595
PS 32:1	0.7326	0.1436
PS 34:1	0.9533	-0.0062
PS 34:2	0.9841	-0.0299
PS 36:1	0.8524	-0.1757
PS 36:2	0.7954	-0.1846
PS 36:3	0.8524	-0.2051
PS 38:1	0.7535	-0.2244
PS 38:2	0.4845	-0.3034
PS 38:3	0.4266	-0.3207
PS 40:3	0.5790	-0.2794
PS 40:4	0.5389	-0.2731
PS 40:5	0.3651	-0.4212
SM 32:1;O2	0.7954	-0.2340
SM 33:1;O2	0.9009	-0.1728
SM 34:0;O2	0.8786	0.0367
SM 34:1;O2	0.8680	-0.1612
SM 35:1;O2	0.8765	-0.1583
SM 36:1;O2	0.9154	0.0115

SM 40:1;O2	0.9154	0.0402
SM 40:2;O2	0.9587	-0.0178
SM 42:1;O2	0.7326	0.1319
SM 42:2;O2	0.8786	0.0384
TG 40:1	0.9154	0.0768
TG 40:2	0.8655	0.0804
TG 41:1	>0.999999	-0.0679
TG 41:2	0.9841	-0.0392
TG 42:1	0.9533	0.0460
TG 42:2	0.9154	0.0455
TG 42:3	0.9533	0.0553
TG 43:1	0.9154	-0.1146
TG 43:2	0.8786	-0.1248
TG 44:0	0.7535	0.1866
TG 44:1	0.8655	0.1023
TG 44:2	0.8786	0.0741
TG 44:3	0.9154	0.0589
TG 45:1	0.9154	0.0077
TG 45:2	0.9319	-0.0671
TG 46:0	0.8383	0.1484
TG 46:1	0.8765	0.0897
TG 46:2	0.8680	0.1088

TG 46:3	0.9154	0.0475
TG 47:1	0.9841	0.0541
TG 47:2	>0.999999	0.0513
TG 47:3	>0.999999	-0.0010
TG 48:1	0.8786	0.1033
TG 48:2	0.8765	0.1000
TG 48:3	0.9009	0.0724
TG 49:1	0.9319	0.0733
TG 49:2	0.9154	0.0612
TG 49:3	0.9841	0.0213
TG 50:1	0.8680	0.1093
TG 50:2	0.9009	0.0817
TG 50:3	0.9009	0.0656
TG 50:4	0.9154	0.0348
TG 50:5	0.9319	-0.0387
TG 51:1	0.9154	-0.0276
TG 51:2	0.9841	0.0117
TG 51:3	0.9841	-0.0193
TG 52:2	0.8655	0.0768
TG 52:3	0.9587	0.0238
TG 52:4	0.9533	-0.0268
TG 52:5	0.9319	-0.0450

TG 53:2	0.9319	-0.0270
TG 53:3	0.9841	-0.0430
TG 53:4	0.8786	-0.1096
TG 54:3	0.9587	0.0394
TG 54:4	0.9154	-0.0568
TG 54:5	0.8680	-0.1187
TG 55:2	0.8655	0.1016
TG 55:3	0.9841	-0.0398
TG 55:4	0.8655	-0.1522
TG 55:5	0.9009	-0.1324
TG 56:3	0.9154	-0.0513
TG 56:4	0.8383	-0.1329
TG 56:5	0.7244	-0.1642
TG 56:6	0.7535	-0.1467
TG 56:7	0.5790	-0.2765
TG 58:2	0.9587	0.0400
TG 58:3	0.9319	-0.0355
TG 58:5	0.4266	-0.2693
TG 58:6	0.4733	-0.3228
TG 58:7	0.4266	-0.4403

Supplementary table 4. Sequences of primers used for mitochondrial DNA analysis.

Primer name	Sequence
16F R	5’-ATAGCGGCTGCACCATCGGGA-3’
16S F	5’-GGGGCGACCTCGGAGCAGAA-3’
APP F	5’-TGTGTGCTCTCCCAGGTCTA-3’
APP R	5’-CAGTTCTGGATGGTCACTGG-3’
B2M F	5’-TGCTGTCTCCATGTTTGATGTATCT-3’
B2M R	5’-TCTCTGCTCCCCACCTCTAAGT-3’
CYTB F	5’-GCCTGCCTGATCCTCCAAAT-3’
CYTB R	5’-AAGGTAGCGGATGATTCAGCC-3’
DLOOP F	5’-CATCTGGTTCCTACTTCAGGG-3’
DLOOP R	5’-CCGTGAGTGGTTAATAGGGTG-3’
HBB F	5’-CAGGTACGGCTGTCATCAGTTAG-3’
HBB R	5’-CATGGTGTCTGTTTGAGGTTGCT-3’

Supplementary table 5. Sequences of the qPCR primers used for analyzing 3T3-L1 gene expression.

mARSB F	5'-CCACGGGCTCTGGAACAAC-3'
mARSB R	5'-GGTGTCCTTCACTGATTGTCTTC-3'
mB3GALNT1 F	5'-CCCTTCCCAATAGGATGTCACT-3'
mB3GALNT1 R	5'-CGCGCTCGATCACATTGTAGT-3'
mCERS1 F	5'-CCACCACACACATCTTTCGG-3'

mCERS1 R	5'-GCAGGTAAGCGCAGTAG-3'
mCHPT1 F	5'-GGTGGGAGAGTCGCTGTTC-3'
mCHPT1 R	5'-TGCCTCCTCCGTGACTGTAG-3'
mGBA F	5'-GCCAGGCTCATCGGATTCTTC-3'
mGBA R	5'-GAGTGCTCTCGTAACGGCT-3'
mGLB1 F	5'-GCACGGCATCTATAATGTCACC-3'
mGLB1 R	5'-GTATCGGAATGGCTGTCCATC-3'
mGPD2 F	5'-ATGGCGTTTCAAAAGGCAGTG-3'
mGPD2 R	5'-ACGGAGAGAGTCCCAAAACAG-3'
mHEXA F	5'-TGGCCCCAGTACATCCAAAC-3'
mHEXA R	5'-GGTTACGGTAGCGTCGAAAGG-3'
mNEU1 F	5'-GGACCGCTGAGCTATTGGG-3'
mNEU1 R	5'-CGGGATGCGGAAAGTGTCTA-3'
mPDK3-F	5'-TCCTGGACTTCGGAAGGGATA-3'
mPDK3-R	5'-ACCTCTCTCATGGTGTTAGCC-3'
mPLA2G15 F	5'-GGGTAACCAGTTGGAAGCAAA-3'
mPLA2G15 R	5'-TTGTCAATCCAGCAGTCAATGAT-3'
mSUCLA2-F	5'-ACCCTTTCGCTGCATGAATAC-3'
mSUCLA2-R	5'-CTGTGCCTTTATCACAACATCCT-3'
mSUMF2 F	5'-GGCTTCAGCTAGGACATGCC -3'
mSUMF2 R	5'-TCTCTGCCATCTGGAGCGT-3'
mUGCG F	5'-ATGGCCTTGTTCGGCTTCG-3'

mUGCG R	5'-GGTGTAACCGGGTGTAGATGAT-3'

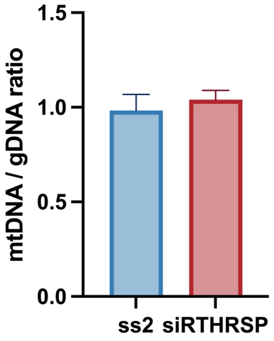

Supplementary fig. 1. Mitochondrial DNA (mtDNA) to genomic DNA (gDNA) ratio, measured by qPCR. THRSP silencing did not alter the ratio (n = 6, two independent experiments).

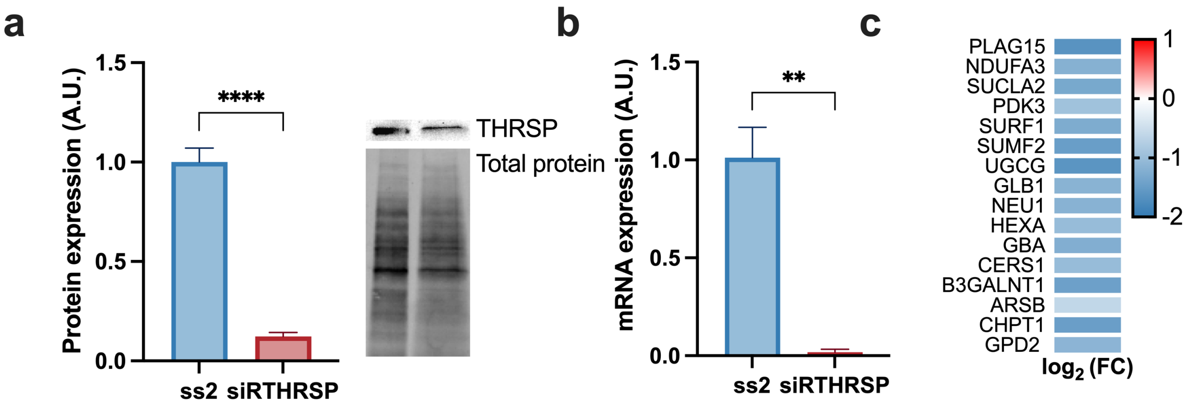

Supplementary fig. 2. THRSP silencing alters the mitochondrial and sphingolipid metabolism genes' expression in 3T3-L1 adipocytes. a-b) To study the impact of THRSP silencing on gene expression in 3T3-L1 adipocytes, the cells were transfected with non-targeting control (SS2) or THRSP siRNA (siRTHRSP; n = 6, two independent experiments). c) Log_2 fold-change (log_2 FC) of mitochondrial function and sphingolipid metabolism associated genes in THRSP-silenced 3T3-L1 adipocytes. All genes were significantly down-regulated ($P \leq 0.05$).

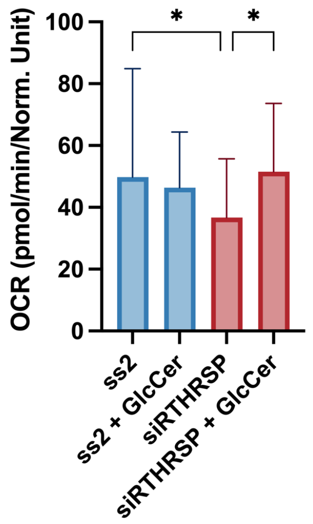

Supplementary fig. 3. Oxygen consumption rate (OCR) is decreased by THRSP silencing (siRTHRSP) in comparison to transfection control (SS2) in SGBS adipocytes but is rescued by addition of glucosylceramides (GlcCer; two independent experiments). The data is represented as mean with SD. Statistical significance is designated as *P ≤ 0.05.
